# A randomised controlled trial investigating the effect of vitamin B12 supplementation on neurological function in healthy older people: the Older People and Enhanced Neurological function (OPEN) study protocol [ISRCTN54195799]

**DOI:** 10.1186/1475-2891-10-22

**Published:** 2011-03-11

**Authors:** Alan D Dangour, Elizabeth Allen, Robert Clarke, Diana Elbourne, Nicky Fasey, Astrid E Fletcher, Louise Letley, Marcus Richards, Ken Whyte, Kerry Mills, Ricardo Uauy

**Affiliations:** 1 Department of Nutrition and Public Health Intervention Research, Faculty of Epidemiology and Population Health, London School of Hygiene & Tropical Medicine, London, UK; 2 Department of Medical Statistics, Faculty of Epidemiology and Population Health, London School of Hygiene & Tropical Medicine, London, UK; 3 Clinical Trial Service Unit, University of Oxford, Oxford, UK; 4 Medical Research Council General Practice Research Framework, London, UK; 5 Department of Non-Communicable Disease Epidemiology, Faculty of Epidemiology and Population Health, London School of Hygiene & Tropical Medicine, London, UK; 6 Medical Research Council Unit for Lifelong Health and Ageing, London, UK; 7 Department of Clinical Neurophysiology, King's College Hospital, London, UK

## Abstract

**Background:**

Vitamin B12 deficiency is common in older people and the prevalence increases with age. Vitamin B12 deficiency may present as macrocytic anaemia, subacute combined degeneration of the spinal cord, or as neuropathy, but is often asymptomatic in older people. The diagnosis and indications for treatment are clear for individuals with low plasma levels of vitamin B12 in the setting of megaloblastic anaemia and neuropathy, but the relevance of treatment of vitamin B12 deficiency in the absence of such clinical signs is uncertain.

**Methods:**

The aim of the present study is to assess whether dietary supplementation with crystalline vitamin B12 will improve electrophysiological indices of neurological function in older people who have biochemical evidence of vitamin B12 insufficiency in the absence of anaemia. To test this hypothesis we designed a randomized double-blind placebo-controlled trial involving 200 older people aged 75 years or greater who were randomly allocated to receive either a daily oral tablet containing 1 mg vitamin B12 or a matching placebo tablet. The primary outcome assessed at 12 months is change in electrophysiological indices of peripheral and central neurosensory responses required for mobility and sensory function. We here report the detailed study protocol.

**Conclusions:**

In view of the high prevalence of vitamin B12 deficiency in later life, the present trial could have considerable significance for public health.

## Background and rationale

Low vitamin B12 status is common among older people [1], and is most frequently a result of age-related gastric atrophy which decreases the production of acid necessary for the release of vitamin B12 from the food matrix, and a decrease in active intrinsic factor which results in vitamin B12 malabsorption [2,3]. Crystalline vitamin B12 is however, relatively well absorbed in most older people [4], and oral supplementation with high-dose crystalline vitamin B12 is recommended to ensure adequate vitamin B12 status in later life [5,6]. Nevertheless, vitamin B12 treatment is widely administered by intramuscular injection in the United Kingdom.

Individuals with vitamin B12 deficiency associated with macrocytic anaemia may present with peripheral neuropathy (sensory disturbances in the extremities such as tingling and numbness, and impaired vibration and joint position sense) and myelopathy that can progress, if untreated, to severe motor problems [5]. The main histopathological finding in vitamin B12 deficiency is a loss of myelin in the white matter of the spinal cord (posterior and lateral columns) and of peripheral nerves, and to a lesser degree in the brain [7]. The mechanism for loss of myelin is not fully understood; while vitamin B12 is required for methylation reactions involved in homocysteine and folate metabolism, vitamin B12 deficiency does not affect the methylation of myelin basic protein [8]. Clinical observations on individuals with pernicious anaemia suggests that intra-muscular treatment with high-dose vitamin B12 over a period of a few weeks typically resolves the neurological symptoms in most cases [9,10], suggesting a reversible biochemical state, and not an irreversible degeneration or loss as seen in some forms of dementia.

There is considerable uncertainty about the relevance of vitamin B12 supplementation for neurological function in older people in the absence of anaemia or macrocytosis. Indeed, there is currently no evidence from randomised controlled trials assessing the effects of dietary supplementation with vitamin B12 on peripheral and central nerve conduction and neurosensory processing. The lack of trial evidence is surprising given that electrophysiological abnormalities associated with vitamin B12 deficiency have been repeatedly demonstrated [11,12]. A single blind before and after study demonstrated improvements, following vitamin B12 treatment, in quantitative electroencephalography in 16 patients [13]. The use of quantitative electroencephalography demonstrates the presence of altered neural function in the absence of clinical symptoms of vitamin B12 deficiency, supporting the concept of using electrophysiological tools to quantify vitamin B12 status.

This is the first randomised controlled trial designed to determine whether supplementation of cognitively normal older people who have low vitamin B12 status in the absence of anaemia will benefit electrophysiological indices of peripheral and central neurosensory responses. Demonstrating that vitamin B12 dependent neurological impairment is present and reversible even in individuals without clinical symptoms could have considerable implications for public health.

## Design and methodology

**Figure 1:**
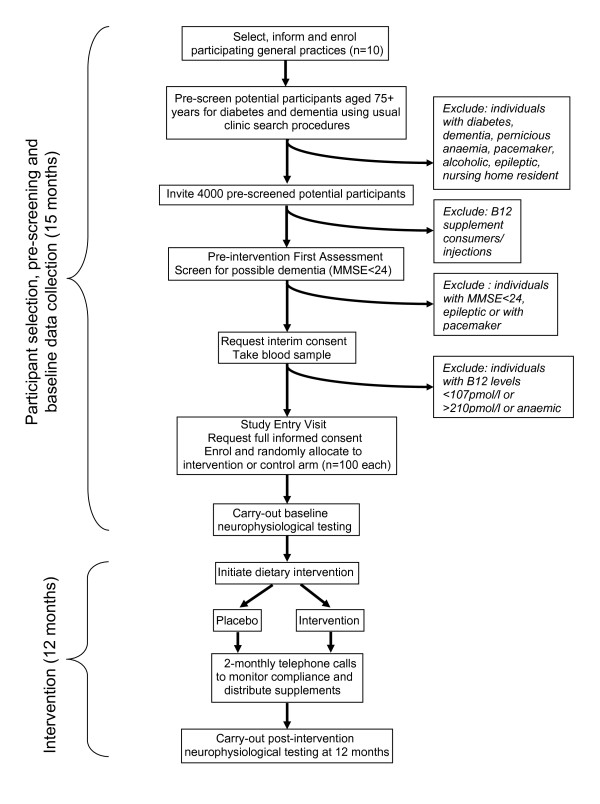
**Flow chart of OPEN study protocol**.

The study is designed as a double-blind randomised placebo-controlled trial. The procedures are illustrated schematically in Figure 1, and detailed in the text. The aim of this trial is to assess whether dietary supplementation with crystalline vitamin B12 will benefit electrophysiological indices of neurological function and cognitive function in older people with biochemical evidence of vitamin B12 deficiency in the absence of anaemia.

Approximately 10 National Health Service general practices, members of the Medical Research Council General Practice Research Framework (GPRF) or the Primary Care Research Network will be recruited. The practices will be situated in the South East of England to facilitate attendance at King's College Hospital. Community-dwelling individuals aged over 75 years of age will be identified from practice registers of participating general practices. The medical records of identified potential participants will be searched and those with pre-existing medically diagnosed diabetes and dementia will be excluded. Individuals with either Type 1 or Type II diabetes will be excluded because of their increased susceptibility for neuropathy that is unrelated to vitamin B12 deficiency. Individuals with pernicious anaemia will be excluded where vitamin B12 treatment is indicated. Individuals known to have alcohol addiction will be excluded, as will individuals with a history of epilepsy or who have implanted metallic devices such as pacemakers (for whom neurophysiological testing is contraindicated). In addition the list of potential participants will be checked by the named General Practitioner in the clinic who will use discretion to exclude individuals considered unsuitable to participate in the study (e.g. recent bereavement, terminal illness).

Potentially eligible individuals identified by this process will each receive a letter and information sheet from their general practice outlining the nature and relevance of the study. The invitation letter will also act as a screen for potential participants who are currently consuming vitamin B12 supplements (either singly or in combination with other vitamins and minerals) or have received a vitamin B12 injection in the last 6 months. Participants reporting the current daily use of vitamin B12 supplements or a vitamin B12 injection within the previous 6 months will be excluded from the trial. Potential participants will be invited to make an appointment with the research nurse at their local general practice. The invitation letter will explicitly state that if they do not wish to participate in the study it will not alter their health care in any way. Non-responders will be sent one reminder three weeks after the initial mailing.

On attending at their general practice, potential participants will be fully informed by the research nurse about the nature and relevance of the study and what will be involved if they agree to take part. The nurse will check with the patient that they do not have a pacemaker, are not epileptic, are not currently taking vitamin B12 supplements on a daily basis and have not had a vitamin B12 injection within the previous 6 months. Potentially eligible participants will be asked if they have been diagnosed with diabetes (confirmed by medical record check), and asked to have an assessment of cognitive function using the Mini Mental State Examination (MMSE) [14]. Participants with an MMSE score of less than 24 (out of a maximum of 30) will be excluded. The cut-off of less than 24 has traditionally been used as a marker for possible dementia in adults. Those with an MMSE score of less than 24 will not be included further, will be thanked for their time and co-operation and will be asked if they wish to make an appointment with their General Practitioner to discuss their MMSE result.

Potential participants without a diagnosis of diabetes and with an MMSE score of 24 or greater will be asked to give full informed consent and to provide a blood sample and, if eligible, have their results and contact information passed to the OPEN study team. Participants providing informed consent will be asked to provide a 4.5 ml blood sample to assess serum vitamin B12 and haemoglobin concentrations.

Research nurses in the participating general practices will provide the results of the blood tests to all potential participants. Eligible individuals are defined as those with a low but not deficient vitamin B12 status who are not anaemic (serum vitamin B12 levels ≥ 107 pmol/l and <210 pmol/l - Beckman Coulter assay [15] and haemoglobin levels ≥ 11 g/l for women and ≥ 12 g/l for men). Individuals found to have above average serum vitamin B12 status (≥ 210 pmol/l - Beckman Coulter assay) will be excluded from the study. Individuals found to have very low vitamin B12 levels (< 107 pmol/l - Beckman Coulter assay) or/and found to be anaemic (haemoglobin concentration <11 g/l for women and <12 g/l for men) will be referred to their General Practitioner for normal practice treatment and excluded from the study.

The contact details of eligible individuals will be passed to the OPEN study manager, who will make telephone contact with participants to restate the nature and relevance of the study and what will be involved if they agree to take part and to invite them to attend a baseline appointment at King's College Hospital. In addition, participants will be asked to complete a questionnaire on diet and psychological health at home and bring it with them to their baseline appointment. Transportation will be provided locally for the participant from their home to the hospital.

At the baseline visit, potential participants will be given the opportunity to discuss the study and ask any questions before being invited to give full informed consent to participate in the study. Individuals giving informed consent will be enrolled in the trial and shown how to contact the OPEN study manager and obtain more information from the study web-site [16].

The OPEN study manger will telephone the central randomisation service to randomise the participant, giving identifying details and the participant's age. Randomisation will allow secure blind allocation of eligible participants to one or other arm of the study. Following random allocation a trial number will be given to each study participant. This will also be used to identify the supply of dietary supplements to be prescribed for each participant (see *Dietary Intervention *below). The study number will be entered on the participant's entry form. Pre-labelled identical-looking packs of dietary supplements will be stored at King's College Hospital. Minimisation criteria will be used to ensure a balance of key prognostic factors using the following two criteria: age group (75-79 and 80+ years) and gender. These two criteria have been selected as there are clear age and gender gradients in vitamin B12 status.

Participants will be asked to complete a questionnaire including a sequence of cognitive function tests, provide a blood sample, and undertake a series of neurophysiological function tests *(*see *Baseline Data Collection *below) which will in total take approximately 75 minutes to complete. Any couples recruited to the study will undertake their assessments separately.

The OPEN study has received ethics committee approvals from the National Research Ethics Service (08/H0305/18) and the London School of Hygiene & Tropical Medicine ethics committee (no. 5298). The OPEN study is registered on ISRCTN (54195799).

## Outcome measures

### Primary outcome

The primary outcome as assessed 12 months after randomisation will be amplitude of tibial motor evoked response. The negative peak amplitude of the peripherally evoked compound muscle action potential reflects the number of motor axons that can be accessed by an electrical stimulus which in turn reflects muscle strength and is therefore a clinically useful parameter in the assessment of treatments which might affect the number or efficacy of motor axons. Lower limb nerves are more affected than upper limb nerves by length dependent axonal dysfunction which is likely to be the case in vitamin B12 deficiency. Compound muscle action potentials (CMAPs) recorded over the abductor hallucis muscle evoked by supramaximal posterior tibial nerve stimuli at the ankle therefore form the primary outcome measure [12,17].

### Secondary outcomes

- Cognitive performance, including tests of memory, executive function and psychomotor speed.

- Functional performance as measured by the timed up and go test.

- Knee and ankle jerk, joint position and vibration sense as clinical markers of neurological function.

- Psychological health as measured by the General Health Questionnaire.

- Peripheral sensory nerve function as measured by superficial peroneal, sural, median and ulnar sensory action potential amplitudes and conduction velocities.

- Peripheral motor nerve function as measured by common peroneal, median and ulnar compound muscle action potential amplitudes; common peroneal, tibial, median and ulnar and tibial conduction velocities; and common peroneal, tibial, median and ulnar minimum F-wave latencies.

- Central motor conduction as measured by cortico-motor threshold, motor evoked potential amplitude, central motor conduction time, and cortical silent period duration in abductor hallucis and abductor digiti minimi muscles.

- Haematological markers.

- Number of hospitalisations for cardiovascular events (myocardial infarction [MI] and stroke) in the12 months after randomisation.

- Death.

## Sample size and recruitment

A sample size of 200 individuals in total (100 per arm) is proposed. With 30% drop-out over 12 months, this would give 90% power to detect at least a 28% change in tibial motor amplitude with 5% significance. Tibial CMAP amplitude is a marker for foot muscle strength and a 28% increase would be associated with clinically relevant improvements in physical coordination and balance. The extent of correlation between measures of tibial motor amplitude between baseline and follow up in the placebo group is currently not known, but if it is high a smaller effect size will be detectable. For example, with an average test-retest correlation across the study of 0.6 and a sample size of 200 individuals, we will be able to detect a 21% change in tibial motor amplitude.

In order to attain this sample size it is expected that approximately 4000 individuals, pre-screened for diabetes, dementia and alcoholism, will be selected from the registers of 10 participating general practices. Experience from a previous study among older people in England and Wales [18] suggests that of the potential participants, 800 (or 1/5 of total invited) will agree to attend a screening appointment. It is estimated that it will take 15 months to recruit participants for the trial. Data from the Banbury B12 Study has been used to define average and low vitamin B12 status [15]. Individuals with serum vitamin B12 concentrations <210 pmol/l (the Banbury B12 study average), and ≥ 107 pmol/l the cut-off for serum vitamin B12 deficiency (Beckman Coulter assay) with no anaemia will be included in the study. We expect that of the 800 attending the screening appointment, approximately 270 individuals will meet the entry criteria for the study.

## Baseline (pre-intervention) data collection

• At home prior to appointment

- Basic data related to diet and alcohol consumption.

- Psychological health assessed using the 30-item General Health Questionnaire (GHQ-30) [19].

• To enable randomization

- Date of birth

- Gender

- Confirmation that no pacemaker or other metallic device is fitted

- Consent

• At baseline visit

- Basic socio-demographic data on age, gender, cardiovascular health (5-year history of hospitalisation for MI or stroke) and educational achievement.

- Cognitive function: simple paper and pencil tests to assess memory and executive function, as well as simple and choice reaction time as markers of psychomotor speed.

- Timed up and go [20] assesses the time taken for a participant to rise from an arm chair, walk 3 metres, turn around and return to sit on the chair and is a good marker of mobility among older people. This may prove an important marker of position sense and gait that are affected in vitamin B12 deficiency.

- Clinical markers of neurological function: presence/absence of knee and ankle jerks, joint position sense and vibration sense in the left and right leg.

- Basic anthropometric measures: height and weight will be measured using standardised procedures in order to enable the calculation of the Body Mass Index (a basic marker of body size) which may be used as a co-variate in analysis.

- Current prescription medication (information provided by General Practice).

- Blood samples to determine baseline status of vitamin B12, holotranscobalamin, total homocysteine and folate, and basic haematological parameters. A sample of blood will be collected into 2 × 5 ml serum separating tubes for subsequent measurement of total homocysteine (tHcy) and holotranscobalamin (holoTC), folate and vitamin B12. This blood sample will be allowed to clot and centrifuge at 2600 rpm for 10 min at a temperature of 4 degrees Centigrade within 2 hours of collection. This sample will be stored at -80 degrees Centigrade prior to laboratory analyses. Serum vitamin B12 will be measured by microbiological assay using *L.Leichmanii *as the growth organism; tHcy concentrations will be determined by an Abbott IMx analyzer, and serum holoTC will be measured using the AXIS-Shield radiommunoassay method [21]. A second blood sample will be collected into a 4 ml Vacutainer tube containing EDTA for haematological parameters (haemoglobin, hematocrit, mean corpuscular volume). Blood samples will be couriered on dry ice in batches from LSHTM to Department of Biochemistry, Trinity College, Dublin, Ireland for analysis of vitamin B12, holoTC, folate and tHcy. Haematological parameters will be assessed using a central laboratory. A sample of DNA will be isolated for each participant and stored for 10 years for future research into potential genetic influences on neurocognitive health in older people.

Obtaining informed consent, randomisation and the collection of baseline information by the OPEN study manager will take approximately 45 minutes. The participant will then undergo neurophysiological testing.

## Nerve conduction velocity and amplitude

### Peripheral nerve conduction

Nerve conduction studies of the right superficial peroneal, sural, common peroneal, tibial, median and ulnar nerves will be performed using standard techniques. All techniques will use surface electrodes and limb temperature will be controlled to be above 28 degrees Centigrade by suitable heating and blankets. Superficial peroneal, sural, median and ulnar sensory action potential amplitude and conduction velocity will be measured. Common peroneal, tibial, median and ulnar motor conduction will be measured by recording from extensor digitorum brevis, abductor hallucis, abductor pollicis brevis and abductor digiti minimi respectively. Nerves will be stimulated supramaximally at proximal and distal sites and conduction velocity in the forearm and distal leg nerve segments calculated. Compound muscle action muscle potential (CMAP) amplitude, distal motor latency and F-wave latency (a measure of conduction times from the distal stimulation site to the spinal cord) will also be measured.

### Central motor conduction

Central motor conduction in the corticospinal tract will be measured by transcranial magnetic stimulation which non-invasively and painlessly excites motor cortex [22]. The time to response in a given muscle is subtracted from an estimate of the peripheral nerve conduction to calculate the central motor conduction time (CMCT). Additionally, from the corticomotor threshold an estimate of cortical inhibitory systems, the cortical silent period, will be obtained.

A magnetic stimulator providing a monophasic pulse and driving a 13 cm diameter circular coil placed over the vertex to excite the hand area of motor cortex will be used. The threshold for excitation will be determined using a standard technique [23]. With the abductor digiti minimi muscle partially activated voluntarily, 8 stimuli at 1.2 times threshold will be delivered to evoke motor evoked potentials (MEPs), the mean amplitude and minimal latency of which will be measured. By using the F-wave latency CMCT will be calculated. Cortical silent period, a measure of inhibitory circuits excited by the stimulus will also be measured. Similarly, using a double cone coil, the leg area of motor cortex will be excited and the MEPs evoked in abductor hallucis also measured. Each participant will receive a maximum of 70 brain stimuli.

The collection of neurophysiological data will take approximately 30 minutes. Participants will need to expose the distal parts of upper and lower limbs. Supramaximal nerve stimuli are routinely used daily to investigate neurological conditions with minimal discomfort. Magnetic brain stimulation causes a minimal sensation like a light tap on the scalp. Participants who are found to have significant neurological deficit will be treated in accordance with standard clinical practice guidelines.

## Dietary Intervention

Following the completion of baseline testing, participants will be introduced to the dietary intervention which will take the form of a daily tablet, identical in size, shape, colour, smell and taste for both the intervention and placebo arms of the trial. The intervention tablets will each contain 1 mg vitamin B12. We have selected this level of daily vitamin B12 supplementation, which is above the established recommended intake, as it has been proven effective in resolving indices of vitamin B12 deficiency in older people [24]. This dose is likely to be necessary to evaluate the effect of subclinical deficit in older people who may have limited absorption of dietary vitamin B12 due to chronic gastritis or defective ileal absorption. Only 1-2% of an oral dose of vitamin B12 is absorbed (by passive diffusion), providing an absorbed amount of 10-20 μg/day [25]. It is also possible that a lower dose may be effective, but the current proposed dose is safe [5] (there is no defined upper limit for vitamin B12), and likely to optimize any potential efficacy.

The OPEN study manager will explain the importance of consuming the supplement every day preferably with a meal, suggesting that it should become part of their daily routine, for example by always consuming the supplement at breakfast time. Participants will be given a 4 month supply of supplements at the baseline hospital visit and will be sent further supplies by post at 4 and 8 months.

## Final participant assessment

After 12 months of intervention, the OPEN study manager will arrange transportation for the participant from their home to King's College Hospital. All participants will be assessed for:

- psychological health: as at baseline;

- cognitive health: as at baseline;

- timed up and go: as at baseline;

- clinical markers of neurological function: as at baseline;

- anthropometry: as at baseline;

- cardiovascular health: record of hospital admissions for MI or stroke between baseline and 12 month appointment;

- haematological indices: as at baseline;

- nerve conduction: as at baseline.

## Trial monitoring

The primary objective of trial monitoring is to ensure that participants adhere to the study regimen, and secondly to minimize drop-out from the study by ensuring frequent contact between participants and the OPEN study manager. Participants will be telephoned by the OPEN study manager soon after the baseline hospital visit to answer any queries and ensure compliance with the trial regimen, and every 2 months throughout the study (6 telephone calls in total). During these calls, participants will be reminded of the importance of the study and of the need to comply with the study protocol.

The OPEN study manager will also record any information a participant volunteers regarding any discomfort caused by the supplement. The recognised side-effects of oral vitamin B12 supplementation include mild transient diarrhoea, and itching. These discomforts are rare at this dosage, generally mild and decrease over time, and the OPEN study manager will provide reassurance to any concerned participants. The 2 month telephone calls will also allow the OPEN study manager to maintain contact with the trial participants. This will allow changes in personal information or ill health and death to be accurately recorded. At the 4 and 8 month telephone calls, the OPEN study manager will arrange for a delivery of a further supply of supplements to the participants home.

## Serious Adverse Events

Participating practices will be asked to report any possible serious adverse events to the OPEN study manager. Any serious adverse events reported by participants to the study manager during 2-monthly follow-up calls will be verified with the General Practice.

## Longer term follow-up

Further follow-up may be the subject of a separate protocol. So that the study organisers do not lose contact with patients should they move address and also to follow up on vital status, including death, participants are being asked to give their agreement for their names and National Health Service (NHS) numbers to be sent to the NHS Central Register.

## Data analyses

Primary analysis will be carried out based upon the groups as randomised ("intention to treat"). Results will be presented as appropriate effects sizes with a measure of precision (95% confidence intervals). Covariates such as gender and baseline age, and vitamin B12, folate and homocysteine status will be adjusted for in the analysis. Further exploratory analysis will be based on those patients who fully follow the treatment protocol.

## Trial organisation

### Investigators

1. Alan Dangour (Principal Investigator PI): public health nutritionist based at the London School of Hygiene & Tropical Medicine (LSHTM), will co-ordinate and manage the overall running of the trial.

2. Elizabeth Allen: medical statistician based at LSHTM.

3. Diana Elbourne: senior trialist based at LSHTM, will co-ordinate activity at the LSHTM Data Coordinating Centre.

4. Astrid Fletcher: senior epidemiologist based at LSHTM.

5. Ricardo Uauy: senior public health nutritionist based at LSHTM.

6. Kerry Mills: Professor of Clinical Neurophysiology responsible for the collection and interpretation of all electrophysiological data based at King's College Hospital.

7. Louise Letley: senior nurse manager for the MRC GPRF with extensive experience of coordinating nurse managed research projects in primary care. Has overall responsibility for the research nurses involved in the study based in General Practices and for the management of quality control within the practices.

8. Robert Clarke: senior clinical epidemiologist based at University of Oxford.

9. Marcus Richards: senior neuropsychologist based at the MRC Centre for Lifelong Health and Ageing, London.

### Project Management Group

A Project Management Group (comprising principal investigators, trial manager, senior research nurse, database manager and statistician) will run the trial on a day to day basis to ensure the smooth operation of the project. Regular review meetings will be held with other members of the team as appropriate.

### Trial Steering Committee

The overall scientific aspects of the project will be managed by a Steering Committee. The Trial Steering Committee comprises expert independent advisors Dr. Edward Reynolds, Consultant Neurologist and Honorary Senior Lecturer, King's College School of Medicine (Chair), Dr. Michael Donaghy, Consultant Neurologist and Reader in Clinical Neurology, University of Oxford, Dr. Amrit Takhar, General Practitioner, Wansford Surgery, Peterborough and lay members Mrs. Ursula Shine and Mrs. Yvonne Davidson [stood down in April 2009].

### Data Monitoring and Safety Committee

An independent Data Monitoring and Safety Committee (DMSC) has been established. The membership comprises Prof. Richard Hughes, Emeritus Professor or Neurology, King's College London (Chair), Prof. Lisette de Groot, Professor of Human Nutrition, Wageningen University and Prof. Graham Dunn, Professor of Medical Statistics, Manchester University. The role of the DMSC is to check on safety by treatment allocation. Although vitamin B12 is not expected to lead to adverse consequences, it is important to monitor safety. In addition, the DMSC will assess compliance data by random treatment allocation and consider data at trial entry by random allocation in order to be able to interpret the compliance and adverse events data. The committee will meet at the start of the trial to agree terms of reference and then at the end of the recruitment period and every 6 months or as agreed.

### Medical Research Council General Practice Research Framework (GPRF)

The responsibilities of the GPRF are to select and support general practices from which the participants are to be drawn. The GPRF will train, monitor and support research nurses throughout the trial, assure recruitment and compliance to the trial protocol.

### Data Co-ordinating Centre

The Data Co-ordinating Centre (DCC) will be based at the LSHTM. The responsibilities of the DCC are to set-up and run systems for data entry, data verification, and to conduct interim and final analyses.

### King's College Hospital

The responsibilities of the investigator at King's College Hospital are to carry out the collection of neurophysiological data at baseline and after 12 months as specified in the protocol, and coordinate with the study manager to ensure that participants appointments are made at a mutually convenient time, interpret primary data and forward cleaned data to the data co-ordinating centre.

### Publication Policy

To safeguard the scientific rigour of the trial, data from this trial will not be presented in public or submitted for publication without permission of the Trial Steering Committee. The requirements for authorship will follow recommended practice in journal guidelines.

### Confidentiality

Data handling will be in accordance with the Data Protection Act. All the research team, including practice staff, will be made aware of the need for complete confidentiality when dealing with patient information. The investigators and local coordinators will ensure conservation of records in areas to which access is restricted.

### Sponsor

The London School of Hygiene & Tropical Medicine will act as the main sponsor for the OPEN study. Delegated responsibilities will be assigned locally.

## Conclusion

Vitamin B12 deficiency is common in older people, and although there are several clinical signs of vitamin B12 deficiency, it can often be asymptomatic. Despite significant interest and scientific rationale in the role of vitamin B12 in nerve conduction, there has to date been no clinical trial investigating the effect of oral vitamin B12 supplementation on electrophysiological indices of neurological function in later life. The current trial is designed to test the hypothesis that daily dietary supplementation with crystalline vitamin B12 will benefit electrophysiological indices of peripheral and central neurosensory responses required for mobility and sensory function. Demonstrating that vitamin B12 dependent neurological impairment is present and reversible even in individuals without clinical symptoms could have considerable public health significance.

## Abbreviations

CMAP: Compound muscle action potential; CMCT: Central motor conduction time; DCC: Data Co-ordinating Centre; DMSC: Data Monitoring and Safety Committee; GHQ-30: 30-item General Health Questionnaire; GPRF: General Practice Research Framework; holoTC: Holotranscobalamin; MEP: Motor evoked potential; MI: Myocardial infarction; MMSE: Mini Mental State Examination; NHS: National Health Service; tHcy: total homocysteine.

## Competing interests

The authors declare that they have no competing interests.

## Authors' contributions

AD and RU conceived the study. AD, EA, RC, DE, AF, LL, KM, MR and RU were applicants for the funding. All authors were involved in designing the study and drafting the protocol. KM designed the electrophysiological testing protocol. MR designed the cognitive testing booklet. All authors read and approved the final protocol.
